# How to Optimize Health Messages About Cancer on Facebook: Mixed-Methods Study

**DOI:** 10.2196/11073

**Published:** 2018-12-18

**Authors:** Priscila Biancovilli, Claudia Jurberg

**Affiliations:** 1 Doctoral School of Health Sciences University of Pécs Pécs Hungary; 2 Laboratório de Imunologia Tumoral Dra. Ottilia Affonso Mitidieri Instituto de Bioquímica Médica Leopoldo de Meis Universidade Federal do Rio de Janeiro Rio de Janeiro Brazil; 3 Fundação Oswaldo Cruz Instituto Oswaldo Cruz Rio de Janeiro Brazil

**Keywords:** cancer, content analysis, Facebook, health, software

## Abstract

**Background:**

Incidence rate of cancer is increasing worldwide, with longer life expectancy being one of the main causes. Yet, between 30% and 50% of cancer cases are preventable, and early detection contributes to a better prognosis. This makes health communication strategies essential. Facebook, the world’s most used social networking site in 2017 and 2018, can be a useful tool for disseminating powerful messages on health promotion, prevention, and early detection.

**Objective:**

We aimed to (1) offer ways of optimizing health messages about cancer on Facebook, focusing on topics, such as risk factors, prevention, treatment, early diagnosis, and cure, and (2) investigate which aspects of these messages generate greater engagement.

**Methods:**

To verify what generates greater engagement in topics related to cancer on Facebook, we analyzed 16 Brazilian pages with the main theme of cancer. We performed a manual analysis of texts, content, and engagement rates. Finally, we developed a software program to operationalize the analysis of Facebook posts. The tool we devised aims to automate the analysis of any Facebook page with cancer as the main theme.

**Results:**

We analyzed 712 posts over a 1-month period. We divided the posts into the following 8 categories: “Testimonies or real-life stories,” “Solidarity,” “Anniversaries,” “Science and health,” “Events,” “Institutional,” “Risk factors,” and “Beauty.” The pages were also organized into groups according to the type of profile to which they belonged (ie, hospitals or foundations, informative, nongovernmental organizations, and personal pages).The results showed that the categories generating greater engagement in Brazil were not those with the highest percentage of cancer-related content. For instance, in the “Informative” group the “Testimonies or real-life stories” category generated an engagement of 79.5%. However, only 9.5% (25/261) of the content within the relevant time period dealt with such topics. Another example concerns the category “Science and health.” Despite being the one with the highest number of posts (129/261, 49.4%), it scored 5th in terms of engagement. This investigation served as the basis for the development of a tool designed to automate the analysis of Facebook pages. The list of categories and keywords generated by this analysis was employed to feed the system, which was then able to categorize posts appearing on a Facebook page. We tested the system on 163 posts and only 34 were classified incorrectly, which amounts to a 20.8% error rate (79.2% accuracy).

**Conclusions:**

The analysis we conducted by categorizing posts and calculating engagement rates shows that the potential of Facebook pages is often underutilized. This occurs because the categories that generate the greatest engagement are often not those most frequently used. The software developed in this research may help administrators of cancer-related pages analyze their posts more easily and increase public interest as a result.

## Introduction

### Background

Cancer is an umbrella term encompassing a group of >200 diseases that have in common the disordered growth of cells invading tissues and organs [1]. The number of cancer-related deaths worldwide increased from 6 million in 2000 to 7.6 million in 2007 [2]. In 2012, there were 8.2 million cancer-related deaths [3], and in 2018, it is estimated that this disease will be responsible for around 9.6 million deaths. While about 1 in 6 deaths globally is due to cancer [4], Brazil has an incidence rate of 205.5 cases of cancer per 100,000 inhabitants, thus ranking tenth in South America and the Caribbean region [3].

For prevention purposes, it is important to reiterate that changes in lifestyle and habits of the population may reduce the likelihood of disease onset. As reported by Anand et al, “Only 5%-10% of all cancer cases can be attributed to genetic defects, whereas the remaining 90%-95% have their roots in the environment and lifestyle. The lifestyle factors include cigarette smoking, diet (fried foods, red meat), alcohol, sun exposure, environmental pollutants, infections, stress, obesity, and physical inactivity” [5]. There is, thus, evidence that prevention is the most cost-effective, long-term strategy for controlling the onset of cancer [6].

In addition to the importance of adopting a healthy lifestyle for prevention, it is crucial to increase early detection in individuals who already exhibit symptoms of the disease. Indeed, when some types of cancer are diagnosed in the early stages, the chances of treatment success and cure (for, at least, 5 years after diagnosis) increase dramatically. According to *Cancer Research UK*, some types of cancer can be treated much more easily if detected early, for example, bowel, breast, ovarian, and lung [7].

### Facebook and Health Communication

Facebook is currently the social networking site with the highest number of active users; in June 2017, it reached 2 billion monthly active users [8]. Every minute, 510,000 comments are posted, 293,000 statuses are updated, and 136,000 photos are uploaded [9]. The most common forms of interaction are reactions (eg, when a user clicks on one of the emojis representing emotions, such as love, surprise, sadness, and anger), comments (eg, when a user writes a text under a post), and shares (eg, when a user shares another person’s post on his or her Facebook profile). Brazil ranks third in the world per the number of Facebook users (130 million), following India (270 million) and the United States (210 million) [10]. Several Facebook pages worldwide are devoted to health promotion. Here, we characterize a “Facebook page” as a public profile created by businesses, organizations, celebrities, or anyone seeking to promote themselves publicly through social media [11].

The active search for health information is associated with greater knowledge of health and with positive behavioral change; that is, individuals tend to become healthier when they are better informed [12]. A number of studies have already explored health-related pages on Facebook to verify the effectiveness of this communication strategy [13-15]. This body of research shows that there is a significant degree of user responsiveness to the topics posted on these pages, suggesting that there is still considerable room for growth in this type of discussion.

### Facebook and Cancer

The use of Facebook as a platform for disseminating health messages focused on cancer treatment, early diagnosis, and prevention has been overlooked in the scientific literature. One of the few papers dealing with this theme [16] analyzed about 13,000 comments posted by visiting users on 3 Brazilian cancer-related pages. It was observed that on these pages there was a strong presence of comments employing religious terms such as “God,” “faith,” “Lord,” “blessed,” “save,” and “pray.” Notably, most of the comments were written by women, and the content of the messages was found to be overwhelmingly positive.

A related study conducted in the United States [12] looked at the National Cancer Institute page to identify the most effective strategies for engaging the audience. The researchers reviewed the posts and comments made on this page and found that “audience engagement is associated with the format of cancer-related posts. Specifically, photo posts received significantly more reactions, comments and shares than videos, links, and status updates (posts that contain only texts)” [12].

Another study published in 2017 [17] implemented a Facebook-based intervention, the main goal of which was to induce users to reduce or stop smoking; the researchers concluded that the interaction between users led to a decrease in the number of cigarettes smoked per week. This result indicates that a Web-based environment of social support and engagement may be beneficial for participants’ health.

Finally, another paper [18] studied the Facebook platform to understand “the most commonly used terms and phrases relating to breast cancer screening and the most commonly shared website links that other women interacted with.” The study concluded that on this social media, women “shared and reacted to links to commercial and informative websites regarding breast cancer and screening”; this result may provide clues for the development of messaging strategies addressing the importance of early detection of breast cancer.

Despite the studies mentioned above, little research is available on the best ways to engage the public in health communication on social media, both in Brazil and worldwide. Academic analyses are even scarcer with respect to cancer-related communication; this might have a negative impact on the Facebook pages of hospitals, nongovernmental organizations (NGOs), and informational organizations, which may end up reaching a lower percentage of the audience than their potential. Therefore, the objectives of this study were to offer ways of optimizing health messages about cancer on Facebook, with special emphasis on topics such as risk factors, prevention, treatment, early diagnosis, and cure, and to investigate which aspects of these messages generate greater engagement in the audience. Notably, the metric of engagement on Facebook is based on the number of reactions, shares, and comments for a post.

## Methods

This study comprised a qualitative and quantitative study [19] with a descriptive purpose [20], not starting from an *a priori* hypothesis.

### Choosing and Organizing Facebook Data

To verify what generates greater engagement in cancer-related topics on Facebook, we analyzed 16 Brazilian pages with the main theme of cancer. In 2017, we studied these pages 2 times over a 1-month period, from March 14 to April 14 and then from April 15 to May 15. With respect to the page selection for this study, we proceeded as follows:

1. We typed the word “cancer” in Facebook’s internal search engine (“câncer” in Portuguese) and selected the “Pages” option.
2. We disregarded pages that were not written in Brazilian Portuguese. We also disregarded pages referring to “Cancer” as an astrological sign. To ensure that the pages were actually Brazilian, we also read the posts to attain better identification of the geographical origin of the page; this was done by either recognizing the way in which Portuguese was written (ie, by looking at the differences between European, African, or Brazilian Portuguese) or seeing that the authors themselves mentioned living in Brazil.
3. To select the pages, we first considered those with a higher number of followers, and then we looked at the updates. Notably, to enter our survey, the page should have, at least, 2 weekly updates in the selected 4-week period. We ended up selecting 15 pages, which were divided into the following categories: personal pages, newsletters, hospitals or foundations, and NGOs.
4. Finally, we analyzed a Facebook page created by us, the purpose of which was to inform the public about the prevention and early diagnosis of cancer. We called this page “Acubens, museu de cancer” (“Acubens, cancer museum” in English).

It is worth noting that in our research, we did not select pages that specifically addressed prevention or early detection. Our intention was rather to identify how Brazilian Facebook pages dealt with cancer-related topics. We include the name of each page, the number of followers in 2017 and a content description in Multimedia Appendix 1.

For our analysis, we used the social media monitoring tool Quintly (quintly.com) because it allows the monitoring of multiple media at the same time, even when a user is not an administrator of the relevant pages. Quintly organizes the publicly available information of all pages (ie, the number of followers, reactions, comments, and shares) in charts and tables, showing, for example, how many new followers a certain page has acquired, or the number of posts created in a selected time period. This service also provides a user with the complete listing of the posts for all the selected pages, collecting the data in a table that indicates the date, time, and type of post. The types of post are sorted into the following categories: photo (any image file), video, event (invitations to events, with the option to accept or decline), status (text-only posts), or link (posts including a Web address redirecting to an external page). These post type definitions mirror those offered by Facebook itself.

### Content Analysis of Posts and Engagement Rate

The analysis of the posts was conducted following the methodology proposed by Bardin [21], which consists of a type of inductive analysis [22]. In our case, 2 researchers performed the analysis independently. We conducted the process of content analysis as follows:

1. *Preanalysis*: It comprised careful and systematic reading of all the text in posts to identify the most relevant categories.
2. *Categorization*: It involved the creation of relevant categories so that all individual posts would fit into, at least, one. In this study, the 2 researchers created their categories independently and subsequently worked together to create a final list. In the case of discrepancy between the 2 initial lists, the 2 researchers discussed the categories concerned until consensus was reached.
3. *Interpretation*: It involved the study of the data and development of inferences [21,23].

After the content analysis process, the 2 researchers created a list of keywords for each category. It was not possible for the same word to feature in more than one category. Moreover, very general words that could fit into any of the categories, such as “cancer” or “chemotherapy,” were not taken into consideration. After the 2 researchers created their lists independently, they met to check similarities and differences and finally a unique list based on consensus was created.

To obtain a more holistic view of the categories, we also established the total impact that each would have, termed as the “engagement rate”. This value considered 3 metrics for each page. We calculated the weighted average reactions, shares, and comments for each post in the 16 relevant pages, assigning a weight of 0.05 for reactions, 0.2 for shares, and 0.75 for comments [23]. The weights created for the calculation of the total engagement took into consideration that the type of engagement (ie, liking, commenting, or sharing) follows a hierarchy according to the amount of effort required by the user to undertake it. For instance, liking a post is usually considered low engagement because it is the simplest and quickest among the 3 available actions, sharing is rather considered a medium form of engagement because a Facebook user identifies with the content to the point that he or she wants to share it on his or her page. Finally, we regard commenting as a high form of engagement; in this case, a Facebook user needs to reflect on the topic in question, draft text, and state his or her opinion publicly.

### Elaboration of a System That Automates the Analysis

Our previous analysis of Facebook pages [23], as well as this study, served as the basis for the development of a tool designed to automate the analysis of any cancer-related Facebook page.

The tool developed constitutes a software program created in JavaScript that allows users to organize different types of Facebook posts according to metrics. While some of these metrics are publicly available (eg, reactions, shares, and comments), others are only accessible by page administrators. The metrics employed by our software are as follows: post reach (how many people viewed that post); post clicks (how many users clicked to read the full text); post hides (how many people hid the page content after reading the post, or reported the page as spam); reactions; shares; comments; engagement (weighted average engagement = number of clicks + reactions × 0.05 + shares × 0.2 + comments × 0.75); and engagement rate (engagement divided by reach). The software then enables the creation of a ranking according to each of these metrics. The ranking can be created by considering all the posts published in a relevant period or by filtering according to the categories to be analyzed.

Moreover, within the software, we created a database of categories and a dictionary of keywords, which were developed by the researchers in an earlier phase of this work; this list is editable, and categories or words may be added or removed at any time. Notably, our system can only “read” complete words, and it does not consider compound or root words. This means that the keywords list contains all the possible variations of a particular word—singular, plural, masculine, and feminine. From these data, the system is then able to tag posts and fit them into categories. If a post uses keywords belonging to more than one category, the system will fit the post into the category exhibiting the highest number of keywords.

Our software is also able to predict the engagement rate that a post would have based on the engagement rates of the previous posts on a given page. More specifically, if a text features keywords that have generated high engagement in previous posts, the likelihood of this new post also having high engagement increases.

## Results

### Content Analysis of Facebook Pages

The 16 Facebook pages that we analyzed produced a total of 712 posts in the relevant 1-month period. As mentioned above, all the pages were organized in groups according to the profile to which they belonged (ie, hospitals or foundations, informative, NGOs and personal pages).

In our previous study [23], we analyzed the texts of 3 Brazilian pages about cancer over a 6-month period (January-June, 2014) and created 8 categories as follows: “Testimonies or real-life stories” (people writing about their experience of cancer or any real-life story); “Solidarity” (posts asking people to make a donation, such as blood or hair); “Anniversaries” (when the main subject of the post was the celebration of some important date); “Science and health” (posts about scientific discoveries, academic research, and progress in treatment); “Events” (when the page administrator organized or publicized some event); “Institutional” (when an institution wrote about itself); “Risk factors” (when the posts addressed habits increasing the risk of cancer, such as smoking); and “Beauty” (posts about makeup, clothes, or hairstyles).

Although we added new pages in this later analysis, we did not have to create new categories with respect to those listed above, indicating that despite the authors and page administrators being different, the spectrum of topics within the theme of cancer remained similar.

The results presented in Table 1 show the analysis of the page performance divided by the following group: hospitals or foundations, informative pages, NGOs, and personal pages.

### Facebook Analytics Software Development

**Figure 1:**
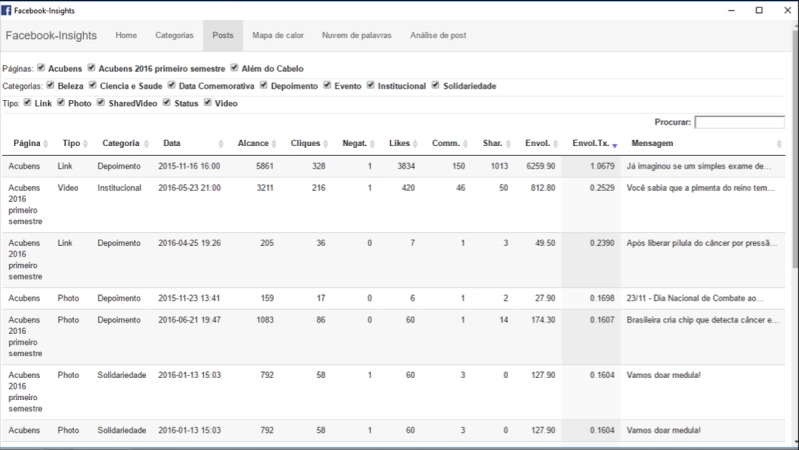
Screenshot of the “posts” tab, displaying the complete list of page posts. (Source: Created by Corbata Informática, 2016).

**Figure 2:**
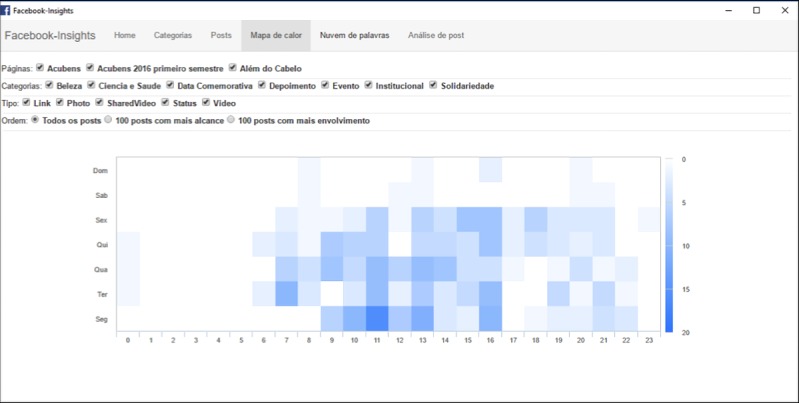
Screenshot of the “Heat map” tab, displaying the days and times of higher engagement on a particular page. (Source: Created by Corbata Informática, 2016).

**Figure 3:**
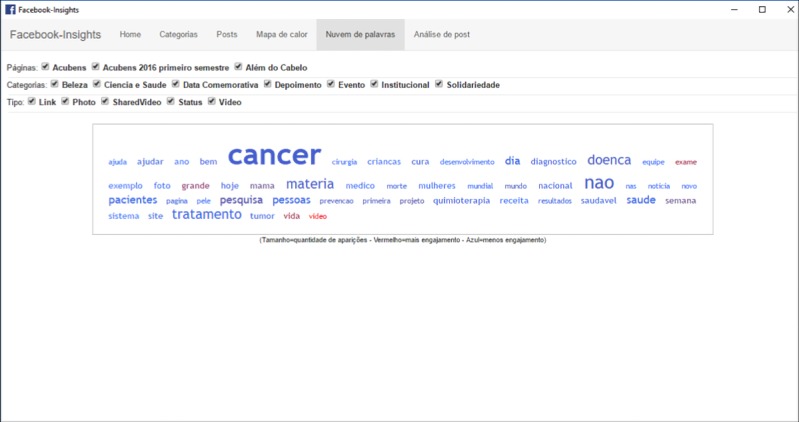
Screenshot of the “Word cloud” tab, showing the words used more frequently on a given page. (Source: Created by Corbata Informática, 2016).

The software we developed for the content analysis of Facebook posts and its classification into categories has a simple and intuitive interface, illustrated in the following Figures 1-3. In Figure 2 darker squares indicate greater the engagement, and in Figure 3 bigger font indicates higher frequency.

Initially, we entered in the software the 8 categories we created, as well as the keywords corresponding to each of these categories. Then, we tested the software through analysis of the page “Acubens, cancer museum,” which was created over the course of 6 months by our research group on the *Oncobiology Program* at the Federal University of Rio de Janeiro. Our goal was to verify whether the tool could actually tag the posts in the correct categories. Over this time period, the page presented 163 published posts. In the first stage of this investigation, 2 researchers categorized all posts manually. Then, the results of the manual classification were compared with that performed automatically by the software. This way, the researchers could verify whether the tool could correctly categorize the posts. Of 163 posts, only 34 were classified in the wrong categories by the tool. This corresponds to an error rate of 20.8% (or 79.2% accuracy). Table 2 summarizes the results of the automated analysis performed by the software and the number of errors found for each category. The errors are deducted from the comparison between the manual analysis done by the researchers and that performed by the software.

The percentage of errors is considered acceptable. Indeed, according to the literature [24-28], the accuracy of multiclass text classification (when texts are classified into ≥3 categories) ranges from 46.9% to 83%.

**Table 1:** Averages of reactions, shares, and comments and weighted average engagement of 16 the pages.

Group		Post, n (%)	Reactions, mean	Shares, mean	Comments, mean	Weighted average engagement
**Hospitals or foundations (n=109)**						
	Solidarity	11 (10)	524	346.9	21.4	111.6
	Anniversaries	0 (0)	N/A^a^	N/A	N/A	N/A
	Institutional	57 (52.2)	825.1	161.3	56.4	115.8
	Testimonies or real-life stories	2 (1.8)	179	26.5	3	16.4
	Science and health	20 (18.3)	1263.3	440.5	47	186.5
	Events	18 (16.5)	283.7	52.5	13.8	35.1
	Beauty	0 (0)	N/A	N/A	N/A	N/A
	Risk factors	1 (0.9)	219	57	12	31.3
**Informative pages (n=261)**						
	Solidarity	19 (7.3)	1400.7	227.9	43	147.9
	Anniversaries	29 (11.1)	2209.9	717.4	27	274.2
	Institutional	26 (9.9)	397.3	72.8	5.7	38.7
	Testimonies or real-life stories	25 (9.6)	1976.5	79.9	108.8	196.4
	Science and health	129 (49.4)	143.4	50.7	3.8	20.1
	Events	28 (10.7)	114.8	28.4	30.4	34.2
	Beauty	1 (0.3)	85	19	3	10.3
	Risk factors	4 (1.5)	76.2	26.5	2.2	10.8
**Nongovernmental organizations** **(n=156)**						
	Solidarity	76 (48.7)	559.8	24.3	16.2	45.0
	Anniversaries	8 (5.1)	1641	108.3	33.8	129.1
	Institutional	27 (17.3)	620.6	37.8	15.7	50.4
	Testimonies or real-life stories	11 (7.1)	505.8	24.5	11.3	38.7
	Science and health	0 (0)	N/A	N/A	N/A	N/A
	Events	33 (21.1)	305.3	84.2	18	45.6
	Beauty	1 (0.6)	124	0	4	9.2
	Risk factors	0 (0)	N/A	N/A	N/A	N/A
**Personal pages (n=186)**						
	Solidarity	53 (28.5)	1885.2	32.8	48.9	135.5
	Anniversaries	9 (4.3)	1320.7	10.8	29.7	90.5
	Institutional	26 (13.9)	340.2	8.7	7.4	24.3
	Testimonies or real-life stories	68 (36.6)	236.6	18.4	26.7	35.6
	Science and health	0 (0)	N/A	N/A	N/A	N/A
	Events	10 (5.3)	401.6	8.8	11.6	30.5
	Beauty	19 (10.2)	149.7	8.3	5.9	16.6
	Risk factors	1 (0.5)	20	1	2	2.7

^a^N/A: not applicable.

**Table 2:** Results of the automated analysis of the page “Acubens, cancer museum” and the number of errors compared with the manual analysis.

Category	Posts analyzed by the tool, n (%)	Errors per category, n (%)
Beauty	1 (0.6)	1 (2.9)
Science and health	95 (57.2)	4 (11.7)
Anniversaries	17 (10.2)	10 (29.4)
Testimonials	25 (15)	14 (41.1)
Events	1 (0.6)	1 (2.9)
Risk factors	11 (6.6)	1 (2.9)
Institutional	6 (3.6)	1 (2.9)
Solidarity	10 (6.0)	2 (5.8)

## Discussion

### Content Analysis of Facebook Pages

In this study, we observed that the categories that generated the greater level of engagement were not those with the highest percentage of posts. For example, in the “Informative pages” group, the “Testimonies or real-life stories” category generated an engagement of 196.4 However, only 9.6% (25/261) of the page content in the period of analysis dealt with such topics. The category with the highest number of posts in the “Informative” group was “Science and health” (129/261, 49.4%); yet, this category was ranked only 6th with respect to engagement.

We observed a similar pattern in the “NGOs” group. While the category generating the greatest engagement was “Anniversaries” (129.1), only 5.1% (8/156) of the page content fell into this category. Within this group of pages, the most frequent category was “Solidarity,” with 48.7% (76/156) of posts. However, the average engagement rate for these posts was 45, around 2.8 times lower than the most successful category and scoring fourth in the average engagement ranking.

Another category with a relatively low presence among the analyzed posts was “Science and health”; this category, along with “Risk factors,” is directly related to topics such as cancer prevention, well-being, and early diagnosis. In the “NGOs” group and on personal pages, nothing was published on the subject. However, in the “Hospital or foundation” group, this category ranked second in terms of average engagement, indicating that people looking for information on hospitals and foundations are more likely to be interested in these topics than people visiting other cancer-related pages. Hence, we suggest that the administrators of hospitals or foundations devote more space to this subject on their Facebook pages.

Furthermore, to increase engagement, it is crucial that the page administrators adopt strategies to incentivize their users to comment more often, as this is the type of participation that demands greater intellectual effort. Given that users who comment invest more time in a post, this is probably the reason why the average number of comments is lower than the average number of shares and reactions across all categories.

Some of the most common strategies used to generate more comments on Facebook consist of asking users questions and responding to all the comments [29]. As Porto emphasized, “The more a user interacts with a particular content producer, the greater the chances of that producer appearing in the user’s news feed” [30]. To increase user engagement, it is, therefore, crucial for the page to encourage similar actions.

### Facebook Analytics Software Development

The category “Science and health” had the largest number of posts (n=95), but it was also the one for which the software committed a small number of errors—only 2. Although the software cannot draw on images or videos that come with the publication, textual analysis proved sufficient for our purposes. In the “Risk factors” category, there was only one error out of 11 posts. An example of text that was correctly classified in the “Science and health” category is as follows:

Cancer can be fought with cell transplantation from healthy subjects. Scientists have discovered that it is possible to fight cancerous tumors by using cells from the immune system of a healthy person and transplanting them in the body of a person with the disease. The research was conducted by the Cancer Institute of the Netherlands and the University of Oslo in Norway and published last week by the journal Science. The researchers noted that by inserting components of a healthy donor's immune system cells into the cells of a patient with cancer in the laboratory, it is possible to get the patient’s body to recognize the tumors and attack them. The research was conducted on 3 patients with melanoma, a type of skin cancer. Read more:http://goo.gl/FgJNvv.

Translated from Portuguese

Although the text contains words belonging to other categories, such as “donor” (“Solidarity”), the software was able to classify the post in the appropriate category, given that most of the words in this section concern “Science and health.”

The classification errors made by the software occurred largely because the tool was not able to analyze the context surrounding a sentence. For instance, the following post was interpreted as “Anniversaries,” despite having been classified as “Institutional” by the researchers:

Any day is a day to break a taboo. Let's talk about cancer. Today's message was recorded with Manoel Gomes and he suggests we see the world in a more positive way. Watch the video by clicking on the link below [link] Get to know @Toda Poesia at [link].

Translated from Portuguese

The mistake arguably happened because the word “day” appears 2 times and it is the only word in this post that also appeared in the keyword list. After this error, we may consider including the word “project” in the “Institutional” category as several publications from “Acubens, cancer museum” in this category contain this word.

### Limitations

With respect to content analysis, one of the limitations concerned the fact that we restricted the study to pages produced in Brazilian Portuguese. We did this out of interest in gaining a better understanding of what is produced on social media about cancer in Brazil and what generates engagement among Brazilians. However, future studies should analyze more broadly the content generated in other countries and languages.

With respect to the software, one limitation concerned the difficulty in choosing the words for each category, as some of them could belong to more than one. In many cases, we had to make choices based on the evaluative criteria of the researchers. However, it may very well be that people with different experiences and writing styles could have classified words in other categories. Another limitation, already mentioned above, may be that the software does not understand the context and, therefore, is unable to capture irony, jokes, ambiguous wording, or figurative language. Moreover, the system is not able to recognize common typing errors.

Despite these limitations, our software could be of help to many research groups and Facebook page administrators wishing to gain a better understanding of what their audience wants and what generates engagement. Other features of the software, such as the “Heat map,” will also be of great value in this process.

### Conclusions

Categorizing posts and calculating engagement rates revealed that the potential of Facebook pages is often underutilized. This may be because the categories generating the greatest engagement are not those used most frequently. In contrast, we have noticed that in some cases, the most attractive category in terms of engagement is among the least published. For instance, it is worth noting that many pages had only a few posts in the “Science and health” category, despite this being one of the most popular. Indeed, along with “Risk factors,” “Science and health” comprises the most relevant categories for public health issues, such as cancer prevention, early diagnosis, and well-being. Given that a high number of cancer cases are related to environmental and lifestyle issues, it is crucial to talk more about prevention and risky behaviors on social media.

However, this study shows that personal pages and the “NGOs” group did not produce any messages about “Science and health.” The “NGOs” group also failed to produce any content on “Risk factors”. Our results suggest that NGOs should include more information about science, health, and risk factors and also set out to promote them more vigorously.

Within the “Hospital or foundation” group, the category “Science and health” was the one that generated the highest weighted average engagement. However, only 18.3% (20/109) of the posts within this group of pages fell into this category. Our suggestion is that page administrators of hospitals or foundations give more space to this subject.

The software developed in this study may certainly help research groups interested in studying cancer-related topics. In addition, the keyword dictionary on cancer could help people who are interested in delving deeper into this topic. Moreover, researchers and groups willing to create new categories and dictionaries could take advantage of our tool to gain a better understanding of what type of content engenders greater engagement among target audiences, thereby collecting information to produce more attractive Web-based content.

## Appendix

Multimedia Appendix 1 Name of each page, number of followers in 2017 and content description.

## Abbreviations

NGO: nongovernmental organization
