# Supplementary material for: How to Optimize Health Messages About Cancer on Facebook: Mixed-Methods Study
Source: JMIR Cancer. 2018 Dec 18;4(2):e11073. doi: 10.2196/11073 (PMC6315252; doi:10.2196/11073)
Supplement: Multimedia Appendix 1 [file cancer_v4i2e11073_app1.pdf]

PERSONAL PAGES		
**Page name**	**Number of followers (August 10th, 2017)**	**Description**
**Além do Cabelo [Besides the Hair]**	15.931	Page of Flavia Maoli, a woman who had Hodgkin's lymphoma twice before she was 30 years old. During her treatment, she shared her routine with her followers. Today, with the treatment completed, she shares her post-treatment routine and publishes positive and motivating messages to help people with cancer live better and enjoy life more.
**Quimioterapia e Beleza [Chemotherapy and Beauty]**	108.938	Page of Flávia Flores, a woman who was diagnosed with breast cancer in 2012. The focus is publications on fashion, beauty, makeup and self-esteem for women with cancer. In 2015, Flávia founded the Chemotherapy and Beauty Institute, an NGO that aims to help people with cancer lead a happier and more meaningful life.
**Minha Vida Comigo [My Life With Me]**	68.275	Page of Vânia Castanheira, a woman who was diagnosed with breast cancer in 2013, at age 31. At the time of the treatment, she shared her routine, her feelings, and gave tips on how to best deal with the disease. Today, with the treatment finished, she has become Medical, Health & Wellness Coach, a type of professional who informs and guides people with cancer to live better, with less fear and more optimism.
**Careca TV [Bald TV]**	2.167.059	Lorena Reginato's page, a 13-year-old girl who became famous after posting videos on YouTube in 2016. Lorena discovered a brain tumor during an MRI scan. On her page, she shares her routine at home, with her family, and at the hospital, as well as asking for donations to help her treat the disease.

INFORMATIVE		
**Page name**	**Number of followers (August 10th, 2017)**	**Description**
**Combate ao câncer [Fight Against Cancer]**	2.193.864	Page created by a medical student and patient son. It aims to inform about prevention, early diagnosis, treatments and cutting-edge research in the oncology area.
**Instituto Oncoguia [Oncoguide Institute]**	214.678	Informational created to publish news on cancer prevention, treatments, new technologies, quality of life and patients' rights.
**Instituto Vencer o Câncer**	63.966	"In 2014, three famous Brazilian oncologists, doctors Antonio Buzaid, Fernando Maluf and Drauzio Varella

**[Institute Win the Cancer]**		accepted the challenge of updating and informing the Brazilian population about advances in cancer treatment". The Facebook page publishes information on health, cancer prevention, early diagnosis and patient rights, among other related topics.
**Acubens, museu de câncer [Acubens, cancer museum]**	8.844	Page created by journalists and scientists of the Oncobiology Program at Federal University of Rio de Janeiro with the objective of deconstructing the negative stigma of cancer, mainly focusing on the young public. It addresses topics such as prevention, risk factors, types of cancer, symptoms, advertising campaigns, early diagnosis and latest science news on the topic. Acubens is the name of the brightest star in the constellation of cancer. The project was considered a museum because the original idea of the project was to disseminate, in a web space, the advances in cancer research and the history of cancer in Brazil and in the world, through internet games, Interactivity and videos. The goal is to show young people how the boundaries are tenuous between aspects of human biology, health and science.
**HOSPITALS/FOUNDATIONS**		
**Page name**	**Number of followers (August 10th, 2017)**	**Description**
**A.C. Camargo Cancer Center**	323.708	A.C.Camargo Cancer Center (private not-for-profit institution) is an integrated center for cancer diagnosis, treatment, teaching and research. Your page addresses cancer risk factors, symptoms, and prevention. In addition, it presents sectors of the hospital, equipment and novelties in the scenario of scientific research.
**Hospital do Câncer de Barretos [Cancer Hospital of Barretos]**	244.869	Located in the state of São Paulo, Brazil, Barretos Cancer Hospital is a national reference in the treatment and prevention of cancer. Its web page is dedicated to the disclosure requests for donations to the hospital, events, and interviews with professionals who work in the hospital, volunteers, patients and reports about the routine of the hospital.
**Instituto do Câncer do Ceará [Cancer Institute of Ceará]**	25.498	Instituto do Câncer do Ceará is a private philanthropic institution dedicated to treatment, teaching and research in the area of oncology in the North and Northeast of Brazil. The page discloses the institute's routine, job openings, events, patient stories and interviews with doctors on aspects of cancer.
**Fundação do Câncer [Cancer Foundation]**	263.653	Cancer Foundation is a non-profit institution created in 1991 that invests in prevention, early diagnosis, programs and projects related to bone marrow and cord blood transplantation, palliative care and research. The page addresses cancer prevention, risk factors, cancer symptoms, information on different types of tumors, requests for donation of money to the foundation and stories of patients overcoming adversity.

**NGOs**		
**Page name**	**Number of followers (August 10th, 2017)**	**Description**
**Cabelegria [Hair and Happiness]**	314.079	The NGO came into being in 2013 with the goal of helping children with cancer, raising donations of hair and making wigs. Currently they have expanded the spectrum of performance and donate wigs to adults as well. The page discloses donation requests, pictures of people who donated hair, images of patients wearing wigs and charity events promoted by the NGO itself.
**Laço Rosa [Pink Loop]**	49.785	Laço Rosa Foundation is a non-profit institution that houses the 1st Online Wigs Bank of Brazil, a project that distributes free wigs and wipes for patients under chemotherapy treatment. The page discloses events organized by the foundation, scientific research on the field of cancer and testimonials, among other subjects.
**Instituto Ronald McDonald [Ronald McDonald Institute]**	937.712	Founded in 1999, Ronald McDonald Institute has the mission to promote the quality of life and health of children and adolescents with cancer. The page shows the actions of the NGO, the routine of children and adolescents in the institute, requests for donations, events and testimonies.
**GRAACC**	258.378	It is an NGO founded in 1991 with the objective of guaranteeing children and adolescents with cancer, within the most advanced scientific standard, the right to achieve all chances of cure with quality of life. The GRAACC hospital has about 3,000 children attended annually. The page mainly discloses events organized by the NGO, testimonies of children and requests for donations.
